# Photo‐Patternable PEDOT:PSS for High Performance Organic Electrochemical Transistors

**DOI:** 10.1002/adma.202521689

**Published:** 2026-03-18

**Authors:** Charles‐Théophile Coen, Niels J. Burghoorn, Jonas G. Hendrikx, Jaap den Toonder, Yoeri van de Burgt

**Affiliations:** ^1^ Department of Mechanical Engineering Microsystems Eindhoven University of Technology Eindhoven The Netherlands; ^2^ Institute For Complex Molecular Systems Eindhoven University of Technology Eindhoven The Netherlands; ^3^ Department of Mechanical Engineering Dynamics and Control Eindhoven University of Technology Eindhoven The Netherlands

**Keywords:** organic electronics, organic mixed ionic‐electronic conductor, photo‐patternable, polymer patterning, semi‐interpenetrating network

## Abstract

Organic mixed ionic‐electronic conductors (OMIECs) have the unique ability to transport both ionic and electronic charges, making them the ideal choice for interaction between biology (ionic) and human technology (electronic). Applied first to sense biological environments, OMIECs are now used to develop brain‐inspired circuits capable of seamlessly “communicating” with biology. However, patterning these organic materials in complex circuits is a challenge as traditional microfabrication techniques are not fully compatible. Current research is still heavily relying on a technique called peel‐off, which involves the physical removal of a sacrificial layer, making it cumbersome to develop highly integrated circuits.

To circumvent these fabrication limitations, a photo‐patternable PEDOT:PSS is developed by blending it with a photo‐sensitive interpenetrating network, allowing features down to 2 µm to be patterned using photolithography on a wide range of substrates. The process fully retains the ability of the OMIEC to transport ions and electrical charges; moreover, organic electrochemical transistors fabricated using this technique outperform the widely used chemically‐cross‐linked GOPS PEDOT:PSS, while having a batch‐to‐batch variability below 5%. Our photo‐patterning process opens the door to monolithic fabrication of miniaturized and highly integrated circuits, allowing to push the limits of interaction between biology and man‐made technology.

## Graphical abstract

Organic mixed ionic‐electronic conductors, such as PEDOT:PSS, are a special class of material capable of dual conductivity, making them a perfect choice to bridge biology and man‐made technology. PEDOT:PSS can be photo‐patterned by blending it with a photo‐sensitive interpenetrating network. The photo‐patterned transistors outperform the GOPS‐cross‐linked PEDOT:PSS due to a higher volumetric capacitance, while showing low batch‐to‐batch variability.

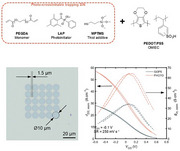

## 1 Introduction

Owing to their ability to transport ions and translate them to electronic charges, organic mixed ionic‐electronic conductors (OMIECs) have been at the core of intense research covering a wide range of fields. [1, 2, 3] While the biocompatibility, functionalizable nature and softness of these materials have been used for traditional bioelectronics (such as sensing and regulating), [4, 5] their ability to mimic neuro‐inspired functions has been leveraged to create artificial synapses and neurons with the goal of developing neuromorphic (brain‐inspired) computing systems, [6, 7] and going toward seamless interaction between biology and electronic technology. [8]

One of the building blocks of many of these applications is the organic electrochemical transistor (OECT). In this three‐terminal transistor, the channel and gate are coupled through an electrolyte, and thanks to the dual conductivity of its OMIEC channel, ions can penetrate the channel upon application of a gate voltage bias. The ions dope/dedope the OMIEC and modulate the channel current. Contrary to field‐effect transistors, the whole bulk of the material is modulated, leading to high transconductance and amplification. [9, 10]

Due to the chemical incompatibility between OMIECs and traditional microfabrication techniques (developed for inorganic materials), a mechanical lift‐off (i.e. peel‐off) is normally used for patterning these materials. [11] This process relies on dry etching two parylene layers (separated by a thin anti‐adhesion layer) followed by spin‐coating the OMIEC and peel‐off of the top layer. Although this process is well‐established and universal in terms of OMIEC choice, its manual nature makes it cumbersome, unscalable, and less reproducible. [12]

These shortcomings can be answered by another patterning method called dry etching. During this process, the spin‐coated OMIEC is covered by either a dual layer (protection layer and a photoresist), [13, 14] or an orthogonal photoresist (fluorinated‐based). [15] These layers are patterned on top of the OMIEC and act as an etch mask. Using reactive ion etching (RIE), the unprotected areas are removed, and the mask can be stripped away.

Although this technique drastically improves the scalability and reproducibility of OECTs, [12] an important drawback remains. Similarly to peel‐off, both these techniques rely on a dry etching step. This is a significant challenge for patterning multiple OMIECs on the same substrate or depositing them on sensitive underlying layers (for example, solid‐state electrolyte, ultra‐thin substrate, or other OMIECs). Either etch stops must be implemented, or etching rates need to be accurately controlled, which considerably complicates design and fabrication, and limits the monolithic integration of OMIECs in complex circuits.

To answer these integration limits, direct photo‐patterning can be used. In this third method, OMIECs are mixed with additives which can either entrap the organic material in a semi‐interpenetrating network (SIN) [16, 17, 18, 19, 20, 21, 22] or directly cross‐link polymer chains of the OMIEC. [23, 24, 25] In both cases, the UV‐exposed parts become insoluble during the development step, emulating the principle of a negative photoresist. This approach solves both the issues of scalability and variability of peel‐off, as well as the complications of physical etching. Although photopatterning of OMIECs has been well investigated with applications ranging from stretchable interconnects for electronic skin, [17, 18] to recording/stimulating electrodes, [18, 20, 21, 22] and mechanical sensors, [21] this method has only been scarcely used for OECTs. [19, 22, 24, 25, 26, 27] Most contributions of OMIEC photo‐patterning either omit to investigate the impact of the photo‐patterning process on the performance of the OECTs or to compare them with the state‐of‐the‐art formulation.

In this work, we introduce a water‐compatible photocross‐linkable SIN capable of micropatterning poly(3,4‐ethylenedioxythiophene) polystyrene sulfonate (PEDOT:PSS). This OMIEC has been one of the most prominent ones to fabricate OECTs thanks to its biocompatibility, processability, and commercial availability. [3] Based on an acrylate monomer, the SIN allows patterning with resolution down to 2 µm using standard lithographic processes on a wide range of substrates, offering a reliable and versatile way of photo‐patterning PEDOT:PSS. The OECTs fabricated using this process show a batch‐to‐batch variability lower than 5% and significantly outperform the conventionally chemically‐cross‐linked PEDOT:PSS transistors with almost double their transconductance, attributed to a higher volumetric capacitance of the former.

## 2 Results

### 2.1 Reliable and Versatile Photo‐patterning of PEDOT:PSS for OECTs

**FIGURE 1:**
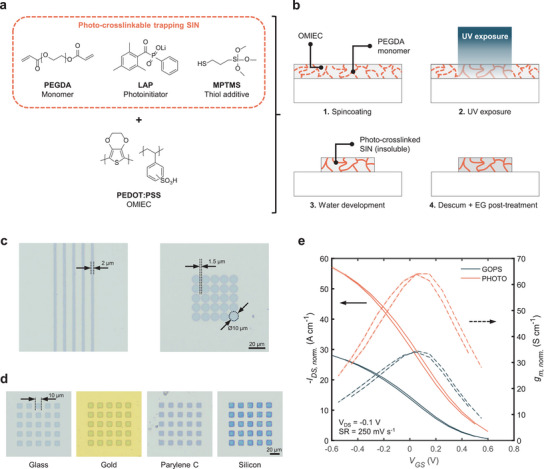
Overview of photo‐patternable PEDOT:PSS. **a)** Chemical structures of the photo‐cross‐linkable SIN. **b)** Schematic of the process flow for photo‐patterning PEDOT:PSS. Under UV exposure, PEGDA monomers cross‐link and entrap the OMIEC, while unexposed parts remain soluble during the development step. The OMIEC's conductivity is then enhanced by an EG post‐treatment. **c)** Optical microscope images of photo‐patterned PEDOT:PSS on glass substrate. Resolution down to 2 µm is achieved using standard i‐line exposure equipment. **d)** Optical microscope images of 10×10 µm^2^ photo‐patterned PEDOT:PSS squares on different substrates. **e)** Transfer curves comparison between photo‐cross‐linked PEDOT:PSS and conventionally GOPS‐cross‐linked PEDOT:PSS in 0.1 m NaCl. Drain current and transconductance are geometry normalized with a factor of *Ld^−1^W^−1^* (*W* = 2000 µm, *L* = 1000 µm). The mean and standard deviation of 10 cycles are shown.

In order to micropattern PEDOT:PSS using UV light, a photocross‐linkable SIN capable of trapping the OMIEC was developed. The network is composed of a water‐soluble monomer poly(ethylene glycol) diacrylate (PEGDA), a photoinitiator lithium phenyl‐2,4,6‐trimethylbenzoylphosphinate (LAP), and a thiol additive (3‐mercaptopropyl)trimethoxysilane (MPTMS) shown in Figure 1a.

The precursor solution is prepared by mixing the chemicals with PEDOT:PSS before being spin‐coated on a substrate (see Methods for more details). The dry film is then exposed to 365 nm UV while being put in contact with a photomask to create the desired pattern. The UV light triggers the radical polymerization of the acrylate groups, which forms the SIN in the exposed parts, rendering them insoluble in water. The unexposed parts, however, remain soluble and can be washed away with water during the development step. Finally, the conductivity of the patterned PEDOT:PSS is enhanced through a post‐treatment using ethylene glycol (EG). [28, 29, 30] This process, illustrated in Figure 1b, is highly reliable, and resolution down to 2 µm can be achieved using standard i‐line exposure equipment (Figure 1c).

During the process, the MPTMS plays the crucial role of radical transferring agent, which allows for efficient radical polymerization in oxygen‐rich environments. [31] Moreover, the additive increases the adhesion of the OMIEC layer to the substrate. The methoxysilane moieties of the MPTMS are, indeed, capable of creating covalent bonds with hydroxyl groups, while its thiol moiety can bind with gold. This allows PEDOT:PSS to be micropatterned on a wide range of substrates, such as glass, gold, Parylene C (PaC), or silicon (Figure 1d).

To evaluate the impact of the process on the properties of the photo‐patternable OMIEC (photo‐PEDOT), such as doping and dedoping, OECTs were fabricated and compared to conventionally chemically‐cross‐linked PEDOT:PSS transistors (Figure 1e), using (3‐glycidyloxypropyl)trimethoxysilane (GOPS). The transfer curve of the photo‐PEDOT‐based OECT shows the typical behavior: a decrease of drain current with positive gate voltages and an increase in drain current for negative gate biasing. [32] An important figure of merit in OECTs is the transconductance (*g_m_* = δ*I_DS_*/δ*V_GS_*), which represents the amplifying factor of the transistor. [33] Interestingly, when scaled with geometry (factor of *Ld^−1^W^−1^*,, with *L* and *W* denoting the channel length and width, and *d* the thickness), the photo‐PEDOT shows almost double the transconductance compared to GOPS‐cross‐linked PEDOT:PSS, reaching above 60 S cm^−1^. This indicates that the photo‐patterning process does not negatively impact the performance of the OMIEC, and could in fact improve certain properties of the material.

### 2.2 Impact of PEGDA Monomer on the Performances of Photo‐patterned OECTs

To understand the effect of the process on the PEDOT:PSS, the role of the primary component of the SIN, the PEGDA monomer, is studied. The influence of the PEGDA load on the properties of the OMIEC is first investigated by choosing an identical molecular weight (MW) of the monomer (700 Da) and by varying its concentration (7 and 10 mg mL^−1^). Other parameters, such as the LAP and MPTMS content, as well as exposure dose, are kept constant.

**FIGURE 2:**
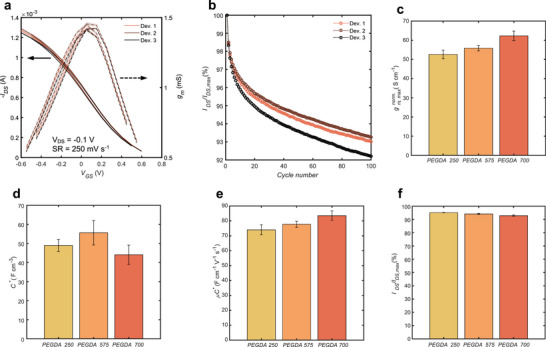
Influence of the PEGDA MW on OECT characteristics. **a)** Transfer curves of 3 photo‐patterned OECT from the same batch (PEGDA 700) in 0.1 m NaCl (*W* = 2000 µm, *L* = 1000 µm). The mean and standard deviation of 10 cycles are shown. **b)** Gate pulsing stability of 3 photo‐patterned OECT from the same batch (PEGDA 700) in 0.1 m NaCl (*W* = 2000 µm, *L* = 1000 µm). Gate pulses of 1 s with a delay of 2 s were set to reach a drain off‐current I_DS_ ≈ 0.1 mA. **c)** Maximum transconductance comparison between different molecular weights of PEGDA measured from the OECT transfer curve in 0.1 m NaCl (*W* = 2000 µm, *L* = 1000 µm). Transconductance is geometry‐normalized with a factor of *Ld^−1^W^−1^*. Bar plot shows mean and 95% confidence interval (*n* = 3). **d)** Volumetric capacitance comparison between different molecular weights of PEGDA measured from PEDOT:PSS films spin‐coated on indium tin oxide (ITO). The volume of PEDOT:PSS is altered by changing the area and thickness of the OMIEC film. Bar plot shows mean and 95% confidence interval (*n* = 6). **e)** µC* comparison between different molecular weights of PEGDA measured from the slope of I_DS_^0.5^ in 0.1 m NaCl (*W* = 2000 µm, *L* = 1000 µm). Bar plot shows mean and 95% confidence interval (*n* = 3). **f)** Gate pulsing stability comparison between different molecular weights of PEGDA measured in 0.1 m NaCl after 100 pulses (*W* = 2000 µm, *L* = 1000 µm). Bar plot shows mean and 95% confidence interval (*n* = 3).

Four Metrics are Obtained from 3 types of Measurements to Compare the Different Concentrations:

1. Transfer curve measurements (Figure 2a) allow monitoring the change in drain current, *I_DS_*, by keeping the drain voltage, *V_DS_*, constant and sweeping the gate voltage, *V_GS_*, at a specific scan rate. From these measurements, the maximum transconductance normalized by the channel geometry, *g_m,max_^norm^* (Equation 1), as well as the mobility of the charge carrier multiplied with the volumetric capacitance, µC^*^ (Equation 2), can be extracted.

(1) $$g_{m,max}^{norm.}=\frac{L}{dW}\;\frac{\delta I_{DS}}{\delta V_{GS}}$$

(2) $$\mu C^{*}=\frac{2L}{dW}{\left(\frac{\delta \sqrt{I_{DS}}}{\delta V_{GS}}\right)}^{2}$$

2. Electrochemical impedance spectroscopy (EIS) is used to independently measure the volumetric capacitance, C^*^, of the OMIEC by using it as the working electrode in a 3‐electrode setup and fitting the impedance spectrum with a Randles circuit. [33] The equivalent capacitor is then normalized by the film volume Equation (3)

(3) $$C^{*}=\frac{C_{EIS}}{dWL}\;$$

3. The stability of the OECT is monitored under a square‐pulse gate by comparing the change in the drain current between two pulse cycles (Figure 2b). The decay of the current over the number of cycles is expressed in Equation (4).

(4) $$Decay=\frac{I_{DS}}{I_{DS,max}}\;$$

Figure S1 shows the comparison of these 4 metrics between the two concentrations of PEGDA 700. From these results, we can conclude that the difference in the content of the monomer does not significantly affect the material properties. It does, however, have an important impact on the photo‐patterning process.

Indeed, increasing the PEGDA load to a high value (such as 10 mg mL^−1^) leads to a visible and detrimental change during the development step. The unexposed parts are no longer water‐soluble and instead delaminate in patches from the substrate (Figure S2a). The subsequently obtained patterns lack sharp edges, and fringes can be observed (Figure S2b). This could be explained by a morphological change of the PEDOT:PSS, such as a decrease of the π–π stacking distance and physical cross‐linking of the PEDOT molecules. [17] However, by decreasing the concentration of PEGDA 700 down to 7 mg mL^−1^, the unexposed parts remain fully soluble during development (Figure S2c) and a sharp outline can be observed after the development step (Figure S2d). The concentration of the monomer must, therefore, be finely tuned as insufficient PEGDA creates an overly fragile SIN that will be washed away during development, and too high a PEGDA concentration induces physical cross‐linking and poor patterning.

It is also important to notice that other chemicals can influence the ability to photo‐pattern PEDOT:PSS, such as the commonly used surfactant dodecylbenzenesulfonic acid (DBSA), as it leads to patch development as well. Moreover, conductivity enhancers, including EG and dimethyl sulfoxide (DMSO), cannot be directly incorporated into the precursor solution as they also induce morphological changes of the PEDOT molecules after spin‐coating. [34]

Although the concentration of PEGDA does not seem to influence the figures of merit of the OECT, the molecular weight of the monomer could have an impact on them. To investigate this effect, three different precursor solutions (Table 1) were optimized with different MW (250, 575, and 700 Da) to yield the best photo‐patternability. Lower MW requires less amount of PEGDA as the acrylate content is higher for a fixed amount of monomer. Interestingly, the ratio between the MW and the PEDGDA load is relatively constant. This indicates that the amount of acrylate moieties necessary for a sufficiently strong interpenetrating network is independent of the PEGDA MW.

**TABLE 1:** Optimized precursor solutions using 3 different PEGDA monomers.

PEGDA MW [Da]	PEDOT:PSS [mL]	PEGDA load [mg]	LAP [mg]	MPTMS [µL]	FS‐30 [µL]
250	1	2	1	2	1
575	1	4.5	1	2	1
700	1	7	1	2	1

Similarly, the same 4 metrics are compared between the 3 molecular weights. To allow a fair comparison, the maximum transconductance is geometry‐normalized with a factor of *Ld^−1^W^−1^*, with *L* and *W* the length and width of the channel, and *d* its dry thickness. Figure 2c shows PEGDA 700 with the highest transconductance of 62.3 ± 2.4 S cm^−1^, slightly higher than PEGDA 575 (55.9 ± 1.4 S cm^−1^) and PEGDA 250 (52.6.0 ± 2.3 S cm^−1^). To explain these results, the hole mobility and volumetric capacitance are compared as the maximum transconductance is related to both quantities. [35]

Although the volumetric capacitances extracted from the EIS measurements (Figure 2d) exhibit the lowest value for PEGDA 700 (44.1 ± 5.1 F cm^−3^), the µC^*^ shows the highest value for PEGDA 700 (83.5 ± 3.2 F cm^−1^ V^−1^ s^−1^) compared to PEGDA 575 (77.7 ± 2.0 F cm^−1^ V^−1^ s^−1^) and PEGDA 250 (74.0 ± 3.4 F cm^−1^ V^−1^ s^−1^), see Figure 2e. The scaling of the µC^*^, therefore, follows a similar trend compared to the transconductance, with maximum values obtained for PEGDA 700, followed by PEGDA 575 and PEGDA 250. The discrepancies between the EIS measurements could be explained by the sensitivity of these metrics to the accurate estimation of geometrical dimensions (Equations (1), (2), (3)). Lastly, Figure 2f shows that the molecular weight does not significantly impact the stability of the drain current under gate pulsing, with decays similar to previously reported. [36]

Overall, we showed that the main metrics of photo‐patterned OECTs are not dependent on the load of the PEGDA monomer, and only slightly on the MW used, with PEGDA 700 showing higher transconductance.

### 2.3 Conventional Fabrication Techniques for OMIEC

Historically, microfabrication techniques like lithography and photoresists were developed for silicon and other inorganic materials, and using them for their organic counterparts has been a well‐documented challenge. [37, 38] To circumvent the incompatibilities between traditional micropatterning processes and organic materials and their solvent, new techniques had to be developed.

**FIGURE 3:**
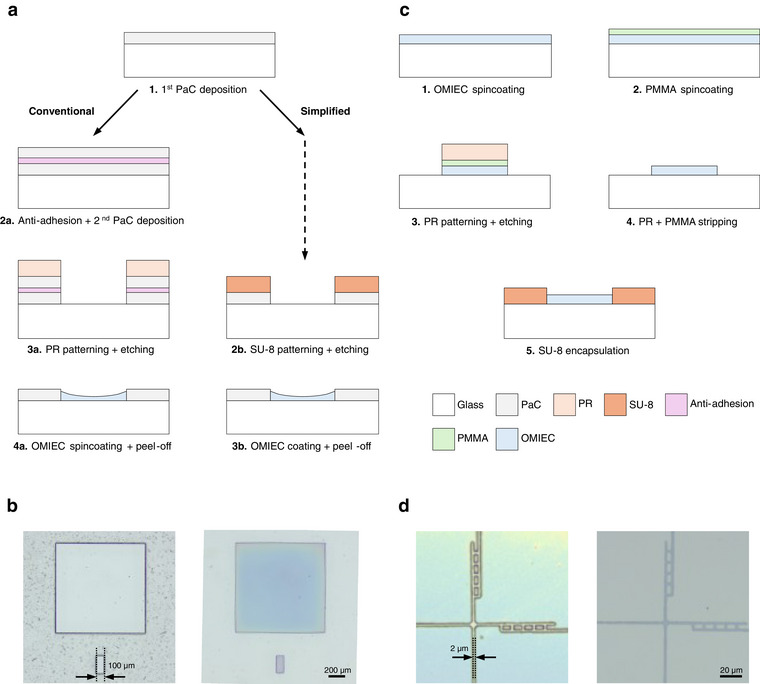
Conventional fabrication methods for OMIEC patterning. **a)** Schematic of a peel‐off process flow. Two PaC layers are conventionally deposited with an anti‐adhesive layer in between and patterned using dry etching. The second PaC layer is peeled off after spin‐coating of the OMIEC (left). A simplified version is developed where a SU‐8 layer acts both as an etch mask and a peel‐off layer. Similarly, the SU‐8 layer is manually peeled off after spin coating the OMIEC (right). **b)** Optical microscope images of the SU‐8 etch mask on top of PaC before etching (left) and of the patterned OMIEC after peeling off (right). **c)** Schematic of the dry etching process flow. The OMIEC is protected by a PMMA layer before being patterned using dry etching. The remaining PMMA and PR are stripped using acetone. **d)** Optical microscope images of the patterned PR on top of PEDOT:PSS before etching (left) and of the patterned OMIEC after stripping (right).

A widely used method for OMIEC film patterning is the peel‐off method, where two PaC layers are deposited with an anti‐adhesive in between, see Figure 3a—left. This double PaC stack is then patterned through dry etching using a photoresist (PR) as an etch mask. Wells created within the stack can be coated with the OMIEC material during the spin‐coating step, before manually peeling off the top PaC layer, leaving the OMIEC inside the wells.

To streamline the peel‐off process and reduce time‐consuming steps, such as PaC deposition, we present a simplified version of it by depositing a SU‐8 layer on top of the first PaC layer, see Figure 3a—right. This epoxy‐based photoresist can be directly patterned and used as an etch mask during the creation of the wells in the underlying PaC. Additionally, thanks to the low adhesion between the SU‐8 and the PaC, the top SU‐8 can be used as the peel‐off layer. Similar to the conventional peel‐off method, the channel and gate of OECTs can easily be fabricated, as shown in Figure 3b, without obvious constraints in terms of choice of OMIECs.

Combining the etch mask and peel‐off layers into one SU‐8 layer allows streamlining the peel‐off process, but one main drawback remains. As the organic materials are deposited inside wells, their thickness is ill‐defined due to the walls present during the spin‐coating (Figure S3b, d). This limits the reproducibility of devices, [12] even within the same batch Figure 3; (Figure S3a, c).

One way to overcome the edge effect present with peel‐off is to pattern OMIECs using dry etching. Figure 3c shows such a process where the organic material is spin‐coated on a flat substrate, before being protected by a PMMA layer. This layer ensures that the subsequent steps do not damage the underlying organic layer or get affected by it. Subsequently, a photoresist is patterned and used as an etch mask during the etching process. Finally, the protective layer as well as the remaining photoresist are stripped away using an organic solvent, such as acetone. This process allows to micropattern OMIECs with resolution down to 2 µm with standard lithography equipment (Figure 3d).

This process is highly robust and can easily be adapted to a wide range of materials, making it a universal solution for micropatterning. However, the use of physical etching to pattern the organic materials is limiting. If the etching rates are not well calibrated, underlying layers or substrates can easily be damaged. This brings an additional challenge for the fabrication of complex circuits combining different materials or fragile underlying layers.

### 2.4 Comparison of Conventional and Photo‐patterned OECTs

To compare the properties of photo‐patterned PEDOT:PSS, two other water‐stable PEDOT:PSS blends are chosen as controls. The first one is the chemically‐cross‐linked PEDOT:PSS blend with GOPS, as it is the most widely used recipe for bioelectronic applications. [39, 40, 41, 42] The second one is a heat‐treated PEDOT:PSS (HEAT), which becomes insoluble in water through phase separation, and has been shown to attain a higher volumetric capacitance compared to GOPS‐cross‐linked films. [43] Both GOPS and HEAT OECTs are fabricated using the peel‐off method to avoid any further processing steps that could potentially affect the performance of the material. The photo‐PEDOT OECTs are photo‐patterned using PEGDA 700 and insulated using SU‐8. Although it has been reported that this permanent photoresist does not affect the electrical properties of PEDOT:PSS, [44] an increase of resistance around 5% is observed after the fabrication of the insulator on top of photo‐PEDOT samples.

To ensure that the results obtained for the photo‐PEDOT are consistent, three different batches of OECT (using different precursor solutions) were initially fabricated to test the reproducibility of the process. The non‐normalized transfer curves of the three batches (Figure S4a) exhibit low variability, which can be attributed to excellent control over the channel thickness when using photo‐patterning (Figure S4b). Figure S4c, d show that device‐to‐device variability remains below 5% (when using a 95% confidence interval), while the batch‐to‐batch variability does not exceed 5% as well when comparing the mean values of the *g_m,max_^norm^* and µC^*^. These results indicate that the photo‐patterning process is reproducible and can be fairly compared to other PEDOT:PSS blends.

**FIGURE 4:**
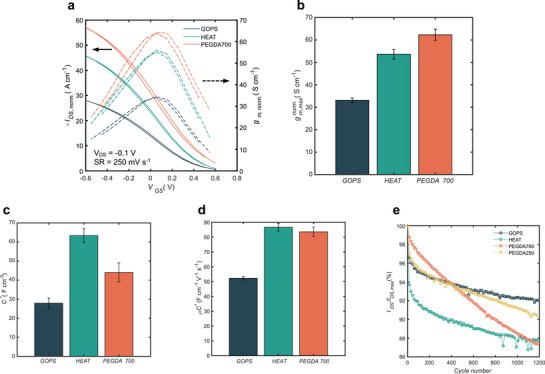
Comparison of OECT characteristics for different PEDOT:PSS blends. **a)** Transfer curve comparison between GOPS‐, HEAT‐ and photo‐cross‐linked (PEGDA 700) PEDOT:PSS in 0.1 m NaCl. Drain current and transconductance are geometry normalized (dry state) with a factor of *dL^−1^W^−1^* (*W* = 2000 µm, *L* = 1000 µm, *d_GOPS_* = 294 nm, *d_HEAT_* = 190 nm, *d_PEGDA700_* = 111 nm). The mean and standard deviation of 10 cycles are shown. **b)** Maximum transconductance comparison measured from OECT transfer curves in 0.1 m NaCl (*W* = 2000 µm, *L* = 1000 µm). Transconductance is geometry‐normalized with a factor of *Ld^−1^W^−1^*. Bar plot shows mean and 95% confidence interval (*n* = 3). **c)** Volumetric capacitance comparison measured from PEDOT:PSS films spin‐coated on ITO. The volume of PEDOT:PSS is altered by changing the area and thickness of the patterned OMIEC film. Bar plot shows mean and 95% confidence interval (*n* = 6). **d)** µC* comparison measured from the slope of I_DS_^0.5^ in 0.1 m NaCl (*W* = 2000 µm, *L* = 1000 µm). Bar plot shows mean and 95% confidence interval (*n* = 3). **e)** Gate pulsing stability comparison measured in 0.1 m NaCl after 1200 pulses (*W* = 2000 µm, *L* = 1000 µm). Gate pulses of 1 s with a delay of 1 s are set to reach a drain off‐current *I_DS_* ≈ 0.1 mA (gate voltage within the range of 0.48 to 0.59 V depending on the blend). Every 15 pulses are plotted for ease of view.

Figure 4a compares the geometry‐normalized transfer curve of the 3 different blends from which the maximum transconductances are obtained (Figure 4b). The geometry‐normalization is done using the amount of material deposited for the channel, i.e. the dimensions in the dry state (for swollen state normalization see Figures S8 and S9). GOPS OECTs show the lowest value with 33.2 ± 1.0 S cm^−1^, while HEAT and PHOTO OECTs exceed these values with 53.7 ± 2.1 and 62.3 ± 2.4 S cm^−1^, respectively. However, one important aspect to notice is the ill‐defined thickness of the GOPS and HEAT samples, attributed to their method of fabrication. With a difference as high as 55% between the center of the channel and its border (Figure S3b, d), properly defining the thickness of these channels is exceptionally difficult, but crucial to correctly characterize OECTs, see Equations (1), (2), (3), which are all highly dependent on an exact representation of the film thickness.

To confirm that the metrics extracted from the peel‐off transistors are in the proper range, GOPS‐cross‐linked OECTs were fabricated using the above‐introduced dry etching method (Figure 3c). These samples show higher reproducibility (Figure S5a) compared to peel‐off ones (Figure S3a), which can be directly associated with their fabrication. The OMIEC is spin‐coated on a flat substrate and then etched away, creating a well‐defined profile for the channel (Figure S5b). Figure S5c, d show that the dry etched samples have slightly higher geometry‐normalized transconductance and µC^*^. This could be explained by an overestimation of the thickness of the peel‐off samples, which is not trivial as the only approach to measure it is to create grooves after the fabrication of the OECT (see Methods for more details). One remarkable difference between the two methods, however, is the final thickness of the channel. While for the peel‐off devices the average thickness is around 311 nm, the dry etched transistors only reach an average value of 179 nm. This difference implies that the similarity between the results attained for the peel‐off and dry etch samples in Figure S5c,d is only possible with the meticulous profile measurement of each transistor. Comparisons with published work that are using the peel‐off method must therefore be made cautiously, as the OMIEC thickness should be individually and carefully measured for every OECT and not extracted from spin‐coated films on a flat substrate Figure 4.

Similarly to before, the hole mobility and volumetric capacitance can be measured to understand the differences observed in the transconductance values of the OECTs. Figure 4c shows the independent measurements of the volumetric capacitance using PEDOT:PSS‐coated ITO samples. As expected, the GOPS‐cross‐linked PEDOT:PSS shows the lowest volumetric capacitance with 27.9 ± 2.7 F cm^−3^ followed by the PHOTO (44.1 ± 5.1 F cm^−3^) and HEAT (63.4 ± 3.6 F cm^−3^) blends. The values obtained for the GOPS‐cross‐linked samples are in the same order of magnitude as previously reported, [10, 35, 45] although the heat‐treated ones are considerably lower. [43] These divergences could again be explained by the edge effect present in peel‐off devices, as reported values are usually measured using peel‐off OECT samples, whereas the values obtained here come from standalone electrode samples (see full fabrication details in the Methods section).

Figure 4d shows the µC^*^ extracted from the transfer curves. The lowest value is observed for the GOPS‐cross‐linked (52.2 ± 1.2 F cm^−1^ V^−1^ s^−1^), followed by the PHOTO‐ (83.5 ± 3.2 F cm^−1^ V^−1^ s^−1^) and HEAT‐cross‐linked (86.6 ± 2.6 F cm^−1^ V^−1^ s^−1^). The stability under pulsing on the gate is shown in Figure 4e. All blends show a sharp reduction of the ON current during the first cycles, followed by a linear decay. GOPS‐cross‐linked PEDOT:PSS shows the best drain current retention with a 2.51% drop over 1000 cycles, while a much larger decay (8.54% over 1000 cycles) is observed for photo‐PEDOT using PEGDA 700. However, when the same stability measurement is performed on the photo‐PEDOT using PEGDA 250, a drop of only 4.31% over 1000 cycles is noted. This indicates that a tighter SIN (PEGDA 250) allows better pulsing stability compared to a looser one (PEGDA 700).

To compare the transient response of the OECTs, a sinusoidal input of increasing frequency is applied to the gate, and the peak‐to‐peak response of the drain current is analyzed. Figure S6b displays a higher cut‐off frequency for photo‐patterned PEDOT:PSS (40 Hz) compared to GOPS‐cross‐linked (32.5 Hz) or HEAT‐cross‐linked (29 Hz) blends. These low cut‐off frequencies are linked to the size of the transistor (*W* = 2000 µm, *L* = 1000 µm), but can be increased by downscaling the OECT's dimensions (Figure S7). [46, 47] Finally the electrical conductivity measurements show similar values for photo‐PEDOT compared to the HEAT blend (Figure S6a), while having a similar ionic conductivity (Figure S6d) and a lower hysteresis (Figure S6c) compared to other traditional formulations.

Overall, the comparison with the GOPS and HEAT recipes shows that photo‐patternable PEDOT:PSS is a viable material for OECTs with significantly higher transconductance compared to the broadly used GOPS‐cross‐linked blend, and having a similar response as the higher performing heat‐treated blend.

## 3 Conclusion

This work introduces a versatile fabrication method to photo‐pattern PEDOT:PSS with micrometer resolution on a broad range of substrates. The SIN used to trap the OMIEC acts as a negative photoresist, where UV‐exposed areas stay on the substrate, while unexposed ones wash away during the development step. Using light to pattern OMIECs films is an extremely reliable and straightforward fabrication method which allows avoiding any incompatibilities with traditional processes developed for inorganic materials. Although photo‐patterning has been used for other organic electronic devices to monolithically fabricate highly integrated circuits, it has hardly been considered for OMIECs.

The OECTs fabricated using the photo‐patternable PEDOT:PSS reach a geometry‐normalized transconductance of 62.3 ± 2.4 S cm^−1^ with a µC^*^ of 83.5 ± 3.2 F cm^−1^ V^−1^ s^−1^. This outperforms the widely used chemically‐cross‐linked GOPS blend which can be attributed to a higher volumetric capacitance of the photo‐PEDOT. Additionally, it is worth noting the inherent difficulty in comparing material's figure of merits across reports. Most of the OMIECs characterization is performed using transistors fabricated with the peel‐off method, even though their ill‐defined thickness makes analysis extremely challenging. It is, therefore, crucial to accurately extract this parameter for better comparison between materials.

Finally, we want to highlight that the SIN developed in this work is tailored to the water‐dispersible PEDOT:PSS. However, the majority of OMIECs are soluble in organic solvent such as chloroform. The community should therefore focus on developing photo‐patterning techniques for other OMIECs than PEDOT:PSS, and study the impact of these processes on the OECT's performance metrics. Finally, in order to fabricate fully solid‐state OECTs, it is essential to develop processes for patterning the electrolyte. [48, 49] This would allow downscaling of devices and monolithic fabrication of organic materials, while increasing the complexity of circuits.

## 4 Methods

Clevios PH1000 (hereinafter referred to as PEDOT:PSS) was purchased from Ossila (M122). PEGDA 250 (475629), 575 (437441), 700 (455008) as well as LAP (900889), MPTMS (175617), EG (324558), silane A174 (440159), PMMA 120k (182230), and diethyl carbonate (D91551) were purchased from Merck. Capstone FS‐30 (1640092‐35‐8) was purchased from ABCR. The photoresist AZ nLOF 2035, developer AZ 726 MIF, and stripper TechniStrip NI555 were purchased from MicroChemicals. The photoresist SU 8–3005 was purchased from Kayakuam, and its developer mr‐Dev 600, from Micro Resist Technology.

### 4.1 Precursor Solution Preparation

#### 4.1.1 Photo‐patternable PEDOT:PSS

Prior to preparing the photo‐PEDOT precursor solutions, PEDOT:PSS is sonicated for 10 min and filtered through a 1 µm glass fiber filter. A 20 mg mL^−1^ LAP in deionized water (DIW) is prepared and used as a stock solution for the photoinitiator.

The precursor solutions are prepared according to Table 1. All concentrations are relative to the volume of PEDOT:PSS. Briefly, the PEGDA is put in a 20 mL vial (2 mg mL^−1^ for PEGDA 250, 4.5 mg mL^−1^ for PEGDA 575 and 7 mg mL^−1^ for PEGDA 700). 1 mg of photoinitiator LAP (i.e. 50 µL of the stock solution) is added per milliliter of PEDOT:PSS. The PEDOT:PSS is then added to the PEGDA and LAP, before incorporating 2 µL of MPTMS and 1 µL of FS‐30 per milliliter of PEDOT:PSS.

The EG solution used to enhance the conductivity of the photo‐patternable PEDOT is made by dissolving EG in DIW to create a final volumetric concentration of 75% EG (typically 1 mL of EG for 0.333 mL of DIW).

#### 4.1.2 GOPS‐cross‐linked PEDOT:PSS

GOPS precursor solution is prepared by first sonicating PEDOT:PSS for 10 min and filtering it through a 1 µm glass fiber filter. PEDOT:PSS is added in a 20 mL vial before adding 60 µL of EG, 10 µL of GOPS, and 1 µL of FS‐30 per milliliter of PEDOT:PSS.

#### 4.1.3 Heat‐treated PEDOT:PSS

HEAT precursor solution is prepared by first sonicating PEDOT:PSS for 10 min and filtering it through a 1 µm glass fiber filter. PEDOT:PSS was added in a 20 mL vial before incorporating 60 µL of EG and 1 µL of FS‐30 per milliliter of PEDOT:PSS.

### 4.2 Adapted Peel‐off Process

The adapted peel‐off process starts with the deposition of a 1.5 µm thick PaC using silane A174 as an adhesion promoter (Specialty Coating Systems PDS 2010 Labcoater). Then a SU 8–3005 is spin‐coated at 1000 RPM for 30 sec and soft‐baked at 95°C for 3 min, before cooling down to room temperature (RT) for 5 min. The photoresist is then exposed to 300 mJ cm^−2^ (SUSS MJB4) through a foil mask (Selba S.A.) designed using the koala python package. [50] Post‐exposure baking is then performed for 20 sec at 95 C, before a 5 min cool down to RT. Development is done in mr‐Dev 600 by soaking 2 times for 15 sec before rinsing the sample with IPA.

The SU‐8 etch mask is now ready, and the underlying PaC can be etched using a RIE (Nordson March RIE 1701). The etching is performed for 4 min at 200 W with a 50 sccm oxygen flow (process pressure: 400 ± 150 mTorr). The sample is subsequently coated with the OMIEC, after which the top SU‐8 layer can be manually peeled off using tape.

### 4.3 Dry Etching Process

The dry etching process starts with the spin‐coating and cross‐linking of the OMIEC (GOPS and HEAT blends were tested). After that a 20 mg mL^−1^ PMMA 120k dissolved in diethyl carbonate is spin‐coated at 4000 RPM for 30 sec and baked at 100°C for 2 min. After being cooled down to RT, AZ nLOF 2035 is spin‐coated at 2000 RPM and soft‐baked at 100°C for 3.5 min. The photoresist is then exposed to 90 mJ cm^−2^ (SUSS MJB4) through a foil mask (Selba S.A.). Post‐exposure baking is then performed for 1 min at 120°C. Development is done in AZ 726 MIF for 50 sec, followed by an extra 10 sec before rinsing the sample with DIW.

The underlying OMIEC is then etched using a RIE (Nordson March RIE 1701). The etching is performed for 30 sec at 300 W with a 20 sccm oxygen flow (process pressure: 250 ± 75 mTorr).

### 4.4 OECT Fabrication

#### 4.4.1 Photo‐PEDOT

Glass substrates (VWR) of 25 mm by 75 mm are sonicated in soap for 10 min, before a 10 min sonication in acetone:IPA (1:1). An AZ nLOF 2035 layer is then spin‐coated at 2000 RPM and soft‐baked at 100°C for 3.5 min. The photoresist is exposed to 90 mJ cm^−2^ (SUSS MJB4) through a foil mask (Selba S.A.). Post‐exposure baking is then performed for 1 min at 120°C. Development is done in AZ 726 MIF for 50 sec, followed by an extra 10 sec before rinsing the sample with DIW. A 10 nm chromium adhesion layer is sputtered before a 100 nm of gold for the interconnects, terminals, and connection pads of the OECTs. The lift‐off process of the AZ nLOF is done in TechniStrip NI555 at 70°C for 30 min, followed by acetone and IPA rinsing.

The photo‐PEDOT precursor solutions (see 4.1.1.) are spin‐coated at 1000 RPM before being air‐dried. The OMIEC is then exposed to 1000 mJ cm^−2^ (SUSS MJB4) through a foil mask (Selba S.A.), before being rinsed with DIW and air dried.

The conductivity of the PEDOT:PSS is then enhanced by spin‐coating 75% EG at 1000 RPM for 30 sec, before baking the sample for 5 min at 180°C. Finally, either a light etching plasma (10 sec at 30 W with a 50 sccm oxygen flow) or an oxygen plasma (1 min at 50 W) is used to descum the sample and get rid of any unexposed PEDOT:PSS. These remaining unexposed parts are too thin (< 5 nm) to be measured using a profilometer and are linked to the incorporation of the surfactant FS‐30 with PEDOT:PSS.

The insulation layer is fabricated by spin‐coating SU 8–3005 at 3000 RPM for 30 sec and soft‐baking it at 95°C for 3 min, before cooling down to (RT for 5 min. The photoresist is then exposed to 300 mJ cm^−2^ (SUSS MJB4) through a foil mask (Selba S.A.). Post‐exposure baking is then performed for 20 sec at 95°C, before a 5 min cooldown to RT. Development is done in mr‐Dev 600 by soaking 2 times for 15 sec before rinsing the sample with IPA.

The OECTs are then immersed in DIW overnight to get rid of any small molecular weight or unreacted chemicals.

#### 4.4.2 GOPS and HEAT PEDOT

The substrates and gold layer are prepared similarly as described above. A PaC and SU‐8 layers are then deposited and patterned as described in section 4.2.

The GOPS and HEAT blends are then spin‐coated at 1000 RPM for 30 sec and dried for 1 min at 100°C, before peeling of the top SU‐8 layer. The GOPS samples are then baked at 120°C for 20 min, and the HEAT samples at 180°C for 2 min, before being rinsed with DIW.

The OECTs are then immersed in DIW overnight to get rid of any small molecular weight or unreacted chemicals.

### 4.5 EIS Sample Fabrication

ITO substrates are cleaned by sonicating them in soap for 10 min, before a 10 min sonication in acetone:IPA (1:1). The PEDOT blends are then spin‐coated at different speeds (700, 1000, and 3000 RPM) before being cross‐linked and rinsed with water. PMMA wells are then taped to create different areas for the EIS measurement (3 by 3 mm^2^ and 4 by 10 mm^2^).

### 4.6 Thickness Measurement

Thickness measurements are performed using a stylus profilometer Dektak (Bruker).

For the photo‐PEDOT OECTs, the thickness of the channel is measured after the EG post‐treatment.

For the peel‐off samples (GOPS and HEAT), 2 trenches are carved above and below the channel using a blade to get the reference of the substrate. Measurements are then performed and averaged between these two trenches.

For the ITO samples, 3 different areas of the samples are scratched with a blade to measure and average the thicknesses of the PEDOT:PSS blend.

### 4.7 Electrical Characterization

The OECT and stability measurements are performed using custom MATLAB scripts to operate a Keithley 2600 (Tektronix).

### 4.8 EIS Measurement

The EIS measurements are performed using a potentiostat SP‐50 (BioLogic) by applying an AC voltage between the working electrode (ITO/PEDOT blend) and the reference electrode (Ag/AgCl). A 10 mV RMS input is applied from 0.1 MHz to 100 mHz and the spectrum is fitted using a Randles circuit.

## Funding

The European Union's Horizon 2020 Research and Innovation Programme, grant agreement no. 802615 and the European Union's Horizon Europe Research and Innovation Programme, grant agreement no. 101125598.

## Conflicts of Interest

The authors declare no conflicts of interest.

## Supporting information




**Supporting File**: adma72778‐sup‐0001‐SuppMat.docx.

## Data Availability

The data that support the findings of this study are available from the corresponding author upon reasonable request.
