# Supplementary material for: Photo‐Patternable PEDOT:PSS for High Performance Organic Electrochemical Transistors
Source: Adv Mater. 2026 Mar 18;38(21):e21689. doi: 10.1002/adma.202521689 (PMC13073073; doi:10.1002/adma.202521689)
Supplement: Supplementary file 1 — Supporting File: adma72778‐sup‐0001‐SuppMat.docx. [file ADMA-38-e21689-s001.docx]

Supporting Information

Photo-patternable PEDOT:PSS for high performance organic electrochemical transistors

Charles-Théophile Coen, Niels J. Burghoorn, Jonas G. Hendrikx, Jaap den Toonder, Yoeri van de Burgt*


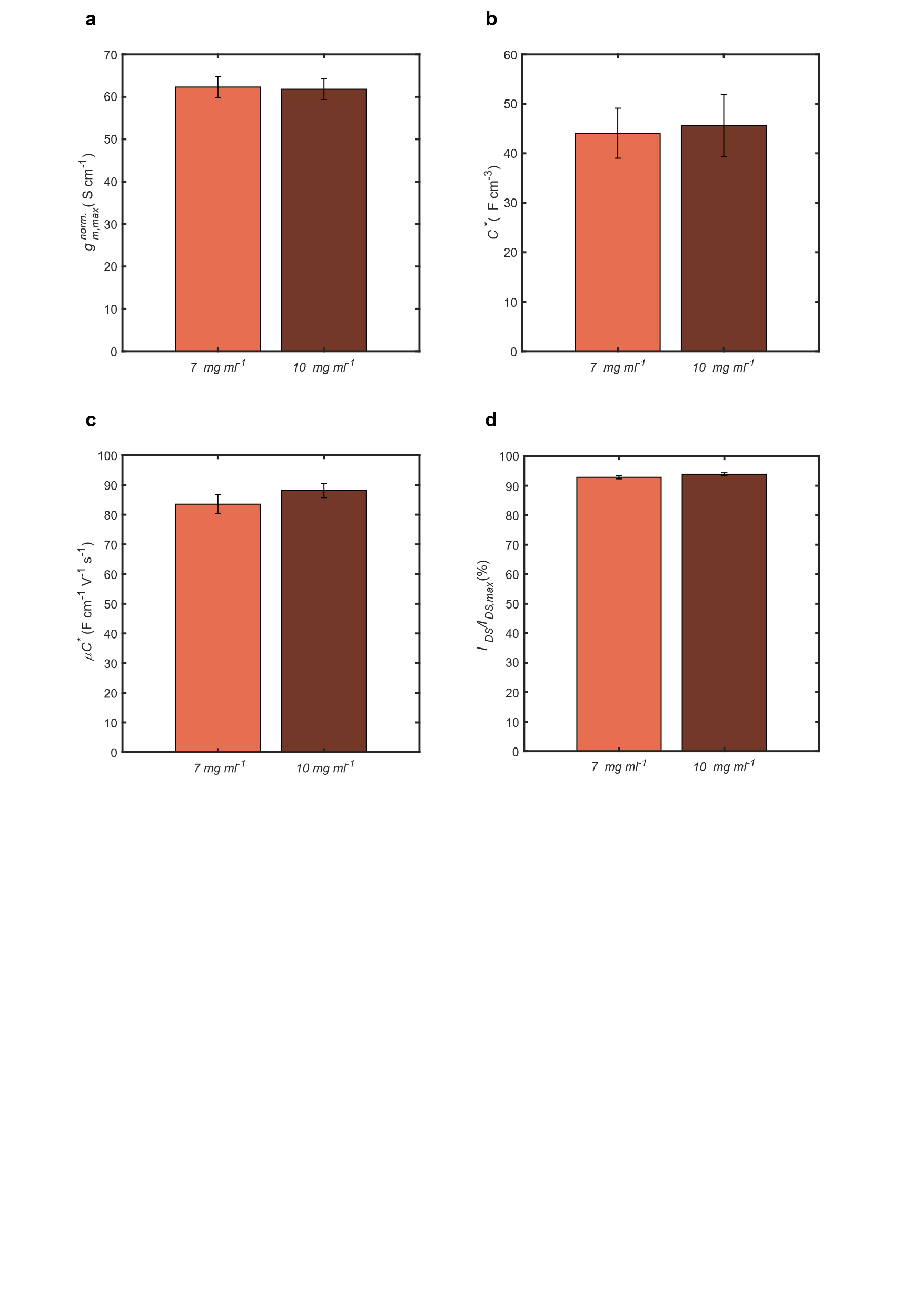


**Figure S1.** Comparison of OECT characteristics for different PEGDA 700 loads in photo-patternable PEDOT:PSS blend. **a)** Maximum transconductance comparison between different load of PEGDA 700 measured from OECT transfer curve in 0.1 M NaCl (W = 2000 µm, L = 1000 µm). Transconductance is geometry normalized with a factor of dL^-1^W^-1^. Bar plot shows mean and 95% confidence interval (n = 3). **b)** Volumetric capacitance comparison between different load of PEGDA 700 measured from PEDOT:PSS films spincoated on ITO. The volume of PEDOT:PSS is altered by changing area and thickness of the OMIEC. Bar plot shows mean and 95% confidence interval (n = 6). **c)** µC* comparison between different load of PEGDA 700 measured from the slope of I_DS_^0.5^ in 0.1 M NaCl (W = 2000 µm, L = 1000 µm). Bar plot shows mean and 95% confidence interval (n = 3). **d)** Gate pulsing stability comparison between different molecular weights of PEGDA measured in 0.1 M NaCl after 100 pulses (W = 2000 µm, L = 1000 µm). Bar plot shows mean and 95% confidence interval (n = 3).


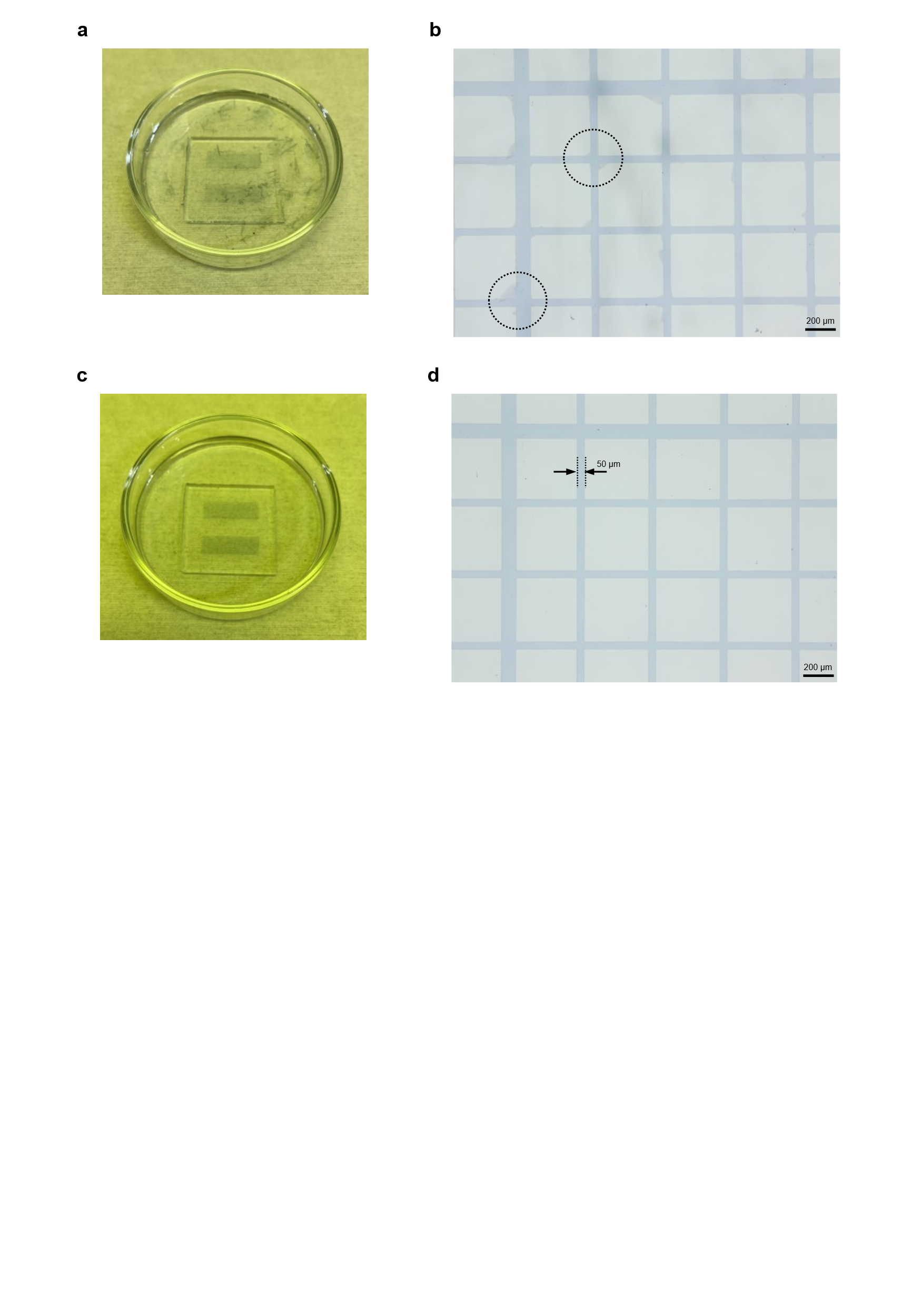


**Figure S2.** Impact of the PEGDA load on the photo-patternable process. **a)** Picture from the development step of photo-patternable PEDOT:PSS (10mg ml^-1^ PEGDA 700) after 20 minutes in water. The unexposed parts are physically crosslinked due to the higher PEGDA load and are not soluble which produces a patch development (unexposed parts only delaminate from the substrate). **b)** Optical microscope image of photo-patternable PEDOT:PSS (10mg ml^-1^ PEGDA 700). The patch development yields ill-defined edges of the pattern, negatively impacting resolution. The circled area show unexposed parts which are physically crosslinked to exposed PEDOT:PSS, creating fringes on the edges. **c)** Picture from the development step of photo-patternable PEDOT:PSS (7mg ml^-1^ PEGDA 700) after a few seconds in water. The unexposed parts are still fully soluble which yields a clean development. **d)** Optical microscope image of photo-patternable PEDOT:PSS (7mg ml^-1^ PEGDA 700). The clean development yields sharp edges, a requirement for optimal resolution.


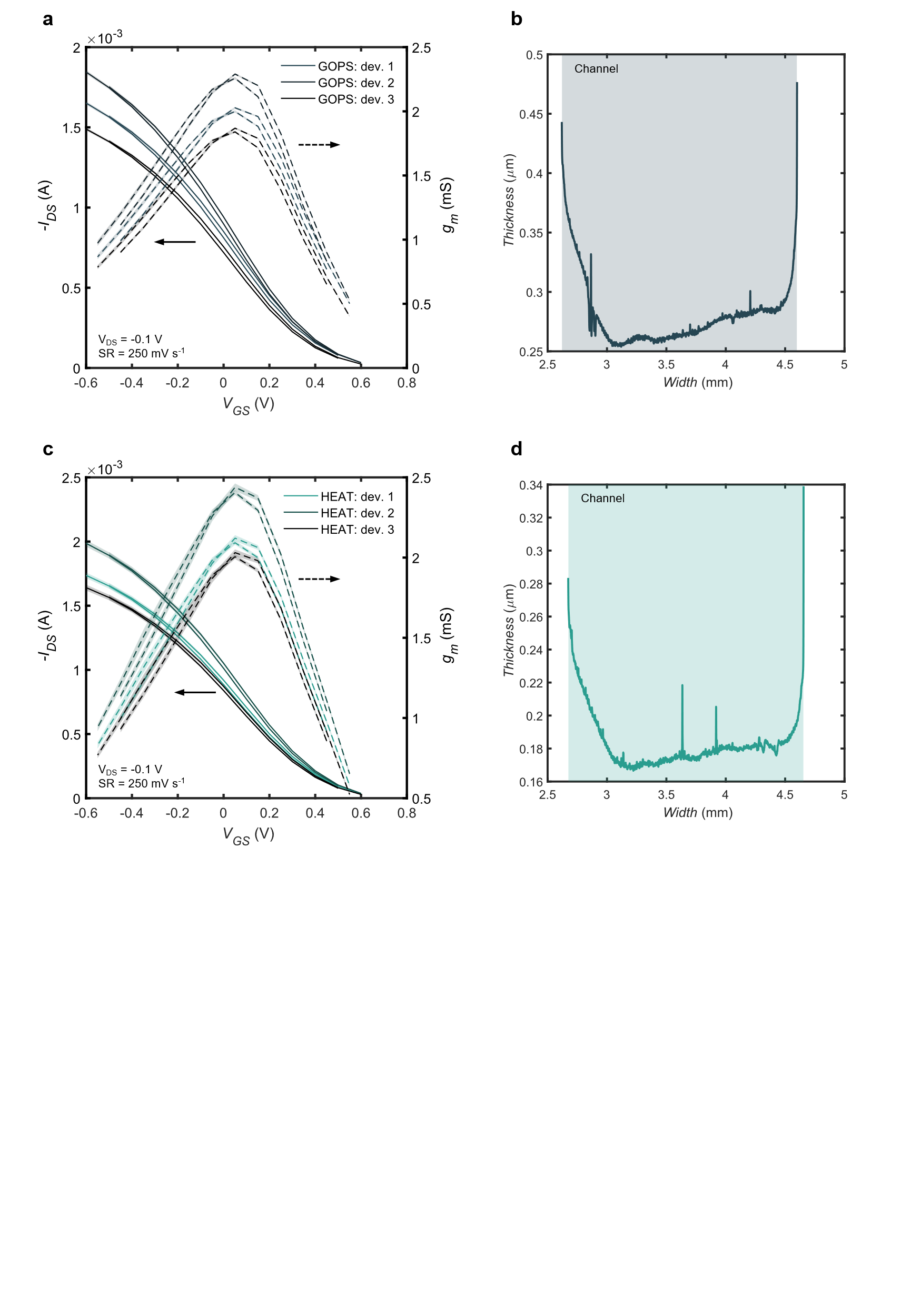


**Figure S3.** Overview of variability in OECT transfer characteristics linked to the uncontrollable thickness of the films. **a)** Transfer curves of 3 GOPS-crosslinked OECT from the same batch in 0.1 M NaCl (W = 2000 µm, L = 1000 µm). Mean and standard deviation of 10 cycles is shown. **b)** Profile measurement along the width of one of the channel of the GOPS-crosslinked OECT. The ill-defined thickness is a direct consequence of the peel-off process, where the OMIEC is spincoated in a well. **c)** Transfer curves of 3 heat treated OECT from the same batch in 0.1 M NaCl (W = 2000 µm, L = 1000 µm). Mean and standard deviation of 10 cycles is shown. **d)** Profile measurement along the width of one of the channel of the heat treated OECT. The ill-defined thickness is a direct consequence of the peel-off process, where the OMIEC is spincoated in a well.


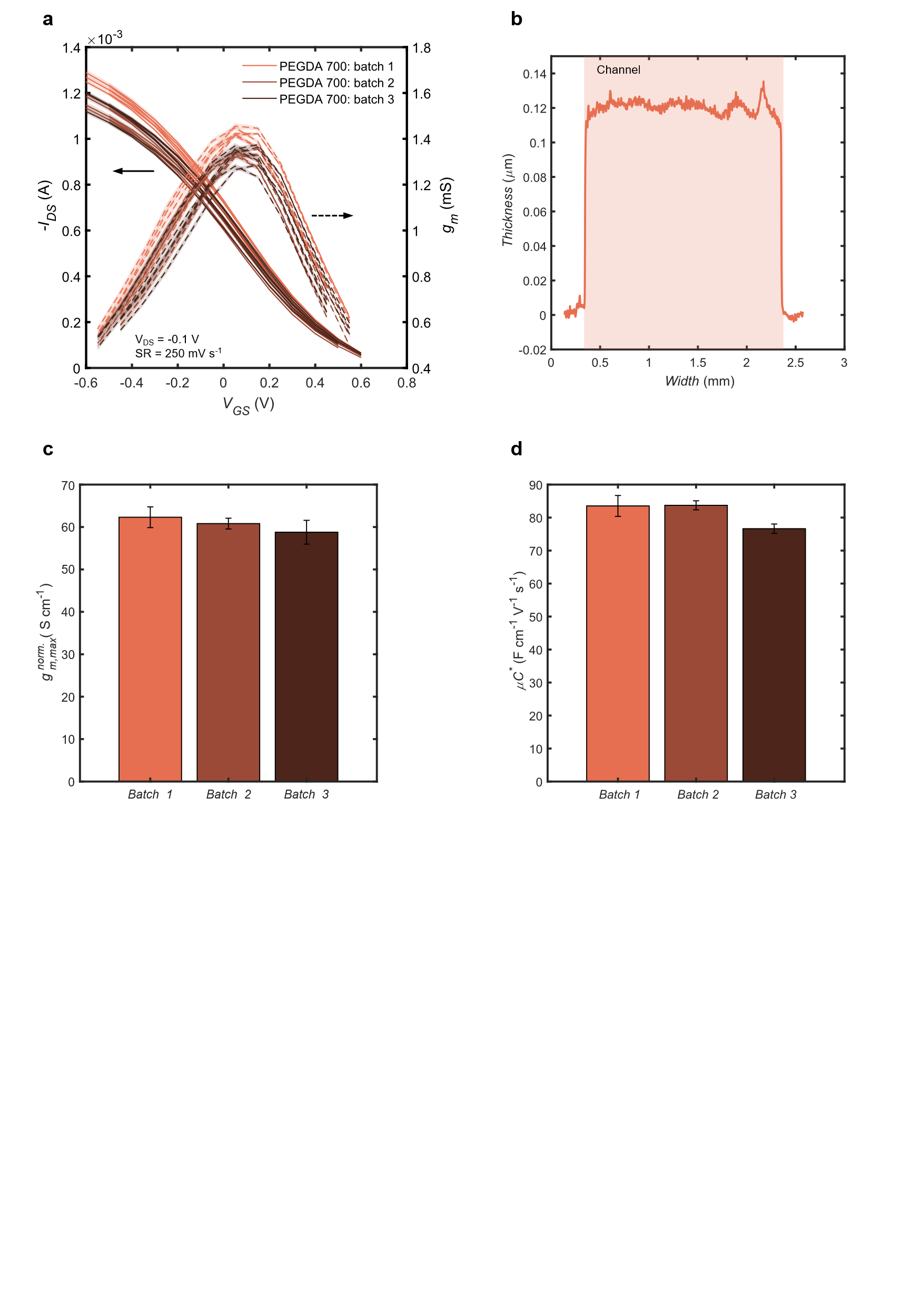


**Figure S4.** Overview of the batch-to-batch variability for the photo-patternable PEDOT:PSS blend. **a)** Transfer curves of 3 photo-patterned OECT from 3 different batch in 0.1 M NaCl (W = 2000 µm, L = 1000 µm). Mean and standard deviation of 10 cycles is shown. **b)** Profile measurement along the width of one of the channel. The photo-patterning process shows a good control over the profile and thickness of the PEDOT:PSS layer. **c)** Maximum transconductance comparison between 3 different batch of PEGDA 700 measured from OECT transfer curve in 0.1 M NaCl (W = 2000 µm, L = 1000 µm). Transconductance is geometry normalized with a factor of dL^-1^W^-1^. Bar plot shows mean and 95% confidence interval (n = 3). **d)** µC* comparison between 3 different batch of PEGDA 700 measured from the slope of I_DS_^0.5^ in 0.1 M NaCl (W = 2000 µm, L = 1000 µm). Bar plot shows mean and 95% confidence interval (n = 3).


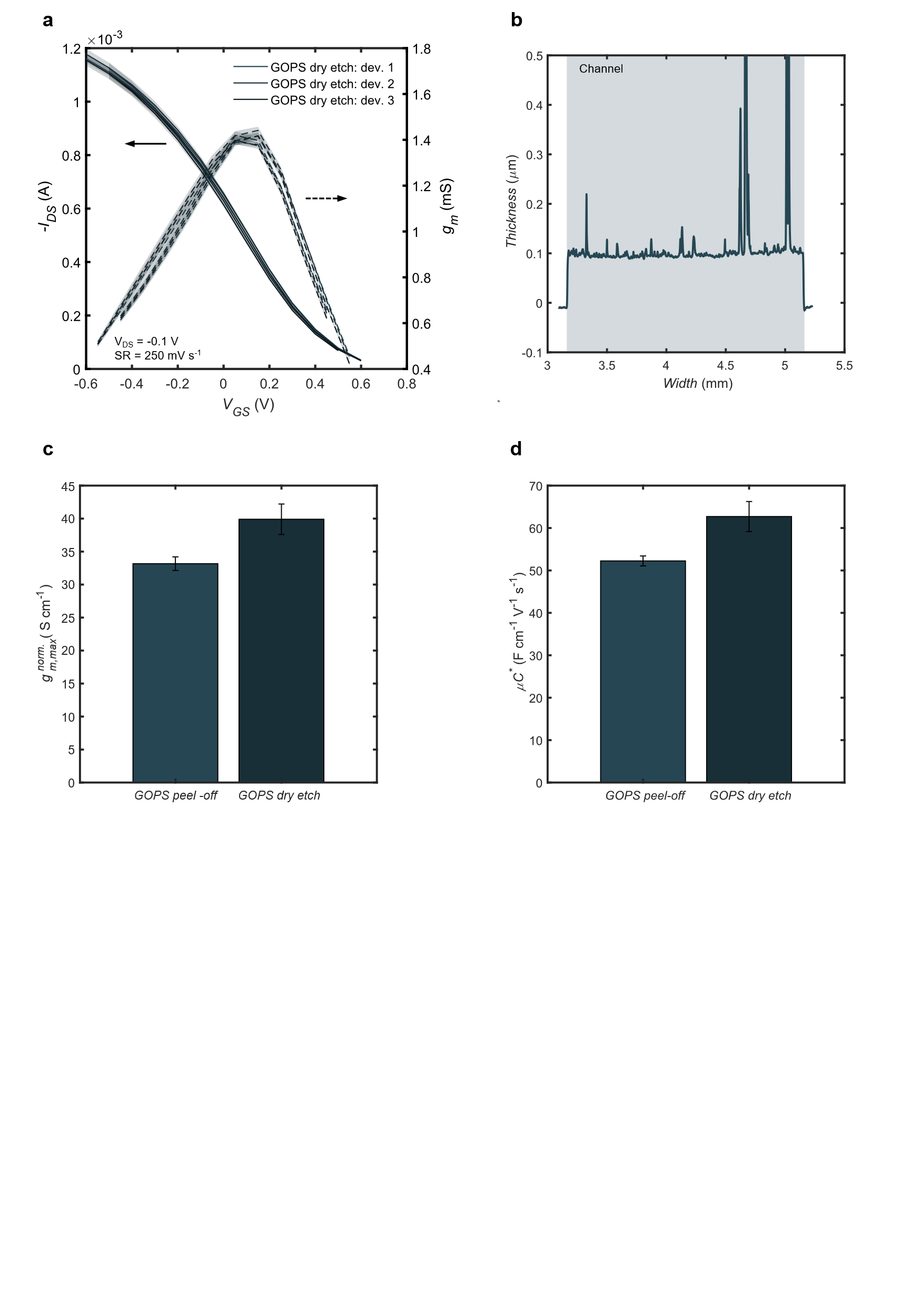


**Figure S5.** Comparison between peeled off and dry etched GOPS-crosslinked PEDOT:PSS blend. **a)** Transfer curves of 3 GOPS-crosslinked OECT from the same batch in 0.1 M NaCl (*W* = 2000 µm, *L* = 1000 µm) fabricated using dry etching. Mean and standard deviation of 10 cycles is shown. **b)** Profile measurement along the width of one of the channel. The dry etching process shows a good control over the profile and thickness of the PEDOT:PSS layer. **c)** Maximum transconductance comparison between peel-off and dry etch GOPS-crosslinked measured from OECT transfer curve in 0.1 M NaCl (*W* = 2000 µm, *L* = 1000 µm). Transconductance is geometry normalized with a factor of *dL^-1^W^-1^*. Bar plot shows mean and 95% confidence interval (*n* = 3). **d)** µC* comparison between peel-off and dry etch GOPS-crosslinked measured from the slope of I_DS_^0.5^ in 0.1 M NaCl (*W* = 2000 µm, *L* = 1000 µm). Bar plot shows mean and 95% confidence interval (*n* = 3).


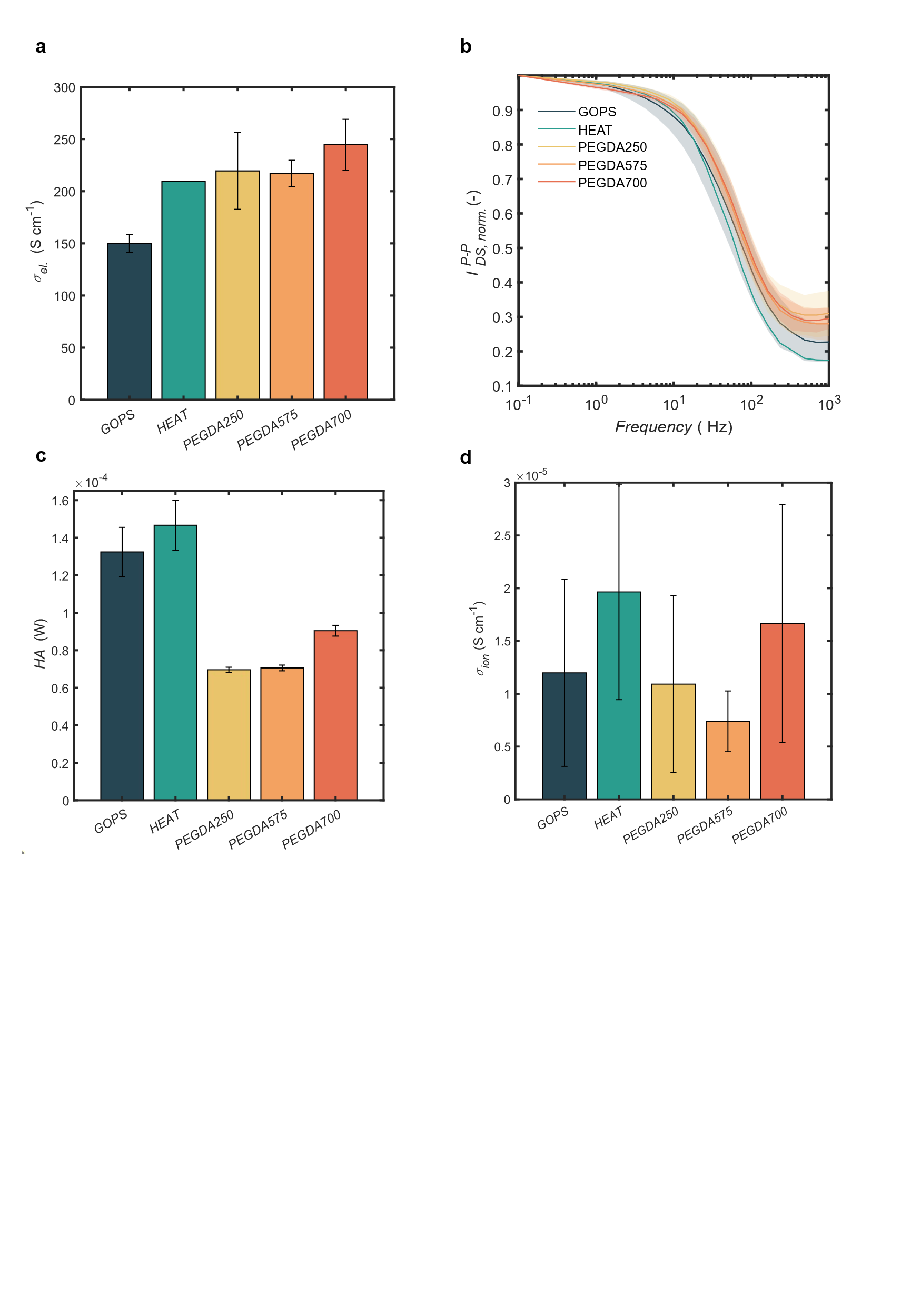


**Figure S6.** Comparison between traditional blend and photo-patterned PEDOT:PSS. **a)** Electrical conductivity using 4-probes measurement. **b)** Frequency response of different OECTs upon a sinusoidal input voltage with an amplitude of 100 mV. The peak-to-peak drain current (*I^P-P^_DS_*) is plotted by normalizing it with the response at low frequency (*I^P-P^_DS, norm._*). **c)** Hysteresis area measured between the forward and backward drain current (averaged over 10 cycles) at 250 mV s^-1^. Bar plot shows mean and 95% confidence interval (n = 3). **d)** Approximate ionic conductivity extracted from the EIS measurements on ITO substrates ($\sigma_{ion}=RWLd^{-1})$. The high variability is attributed to the low thickness, and thus high resistance, of the measured films.


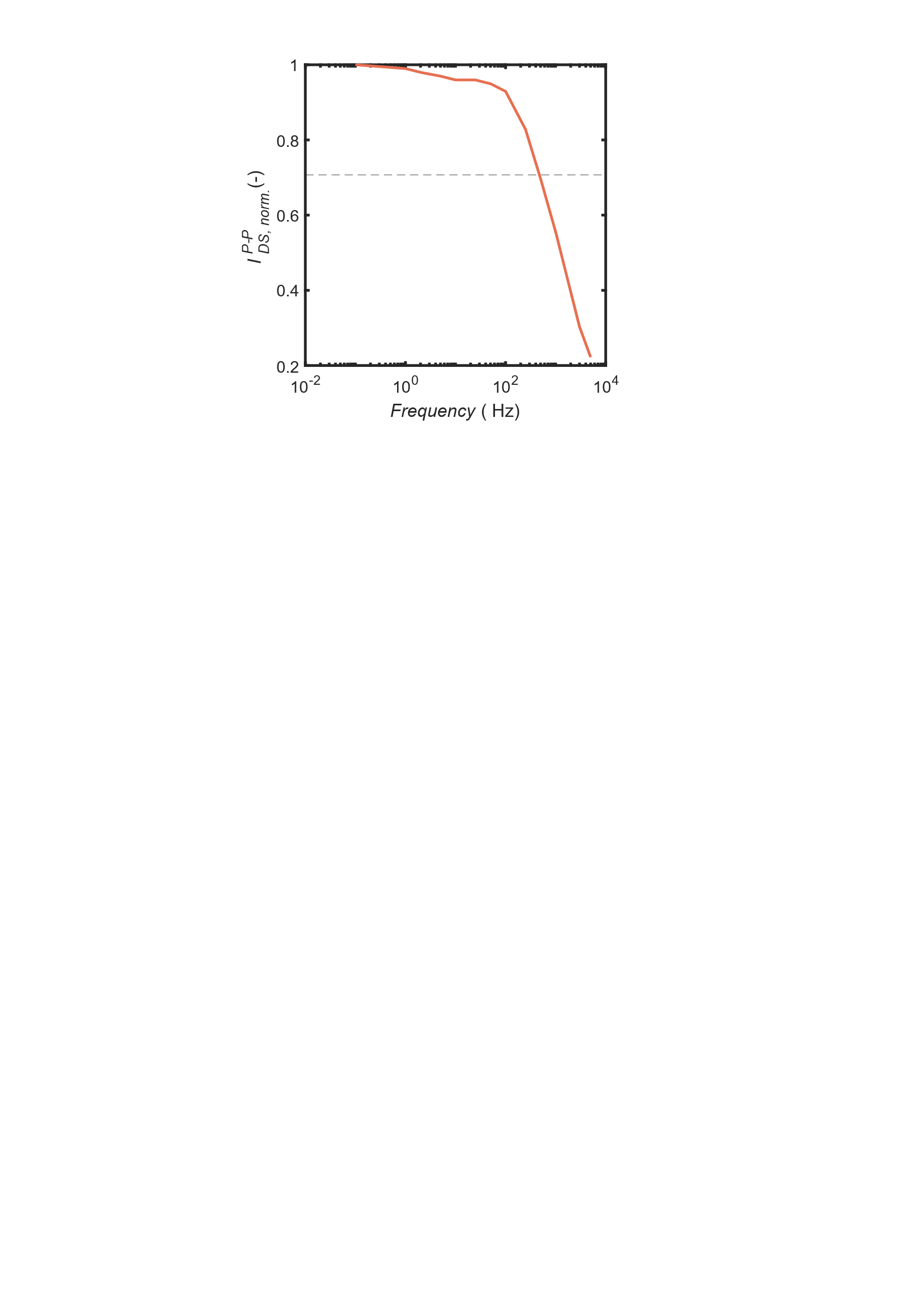


**Figure S7.** Frequency response of a smaller OECT (*W* = 500 µm, *L* = 25 µm) upon a sinusoidal input voltage with an amplitude of 100 mV. The peak-to-peak drain current (*I^P-P^_DS_*) is plotted by normalizing it with the response at low frequency (*I^P-P^_DS, norm._*). This particular OECT exhibits a cut-off frequency around 500 Hz, but can be increased by further downscaling the width and length of the transistor.


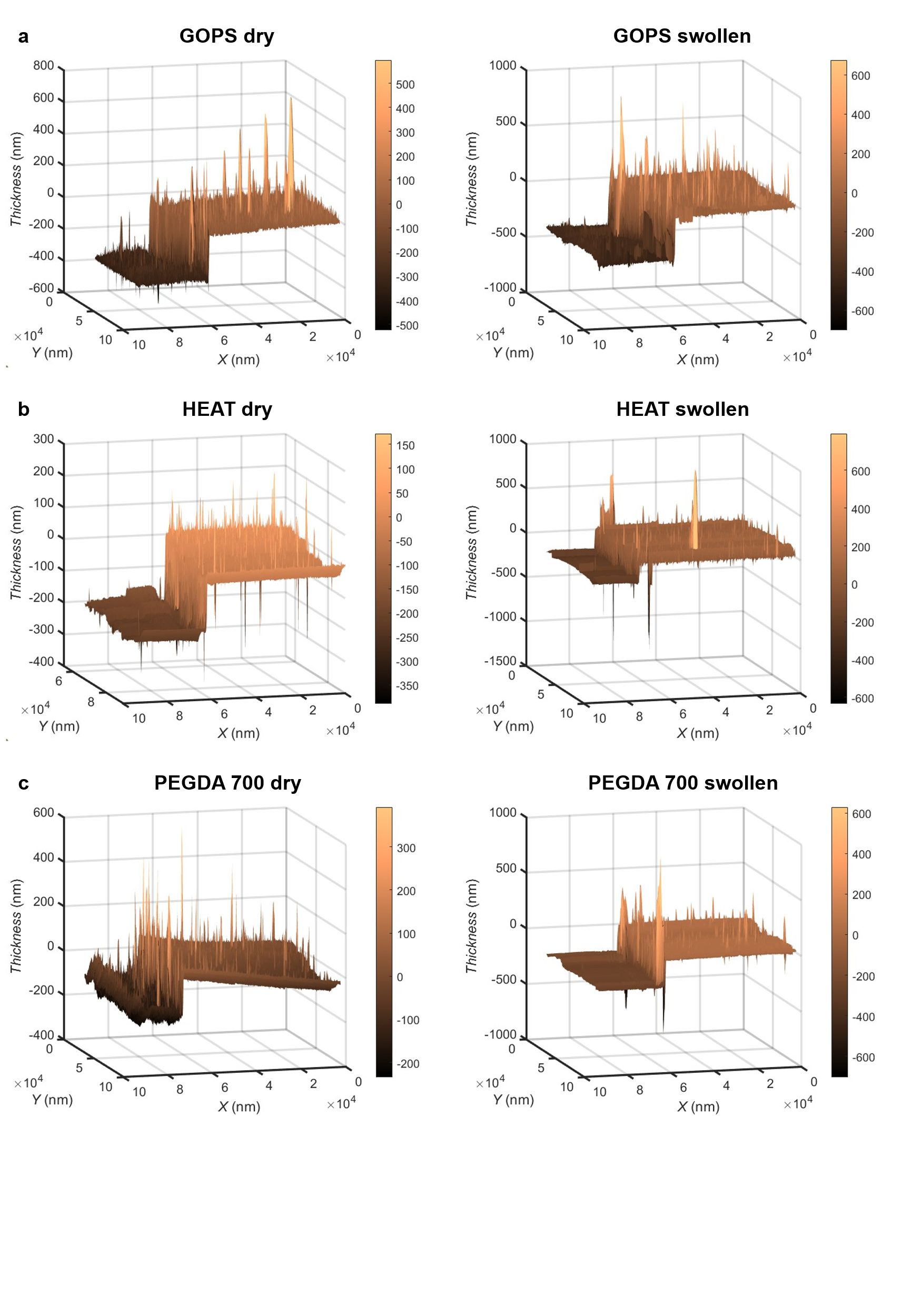


**Figure S8.** AFM measurement between dry and swollen state for different blend of PEDOT:PSS. The swollen state is measured by dropping 0.1 M NaCl before performing the measurement. **a)** GOPS dry (average thickness 320 nm) and swollen (average thickness 384 nm). **b)** HEAT dry (average thickness 190 nm) and swollen (average thickness 245 nm). **c)** Photo-patterned using PEGDA 700 dry (average thickness 210 nm) and swollen (average thickness 268 nm). The swollen ratio obtained are 1.2 for GOPS, 1.29 for HEAT and 1.28 for PEGDA 700.


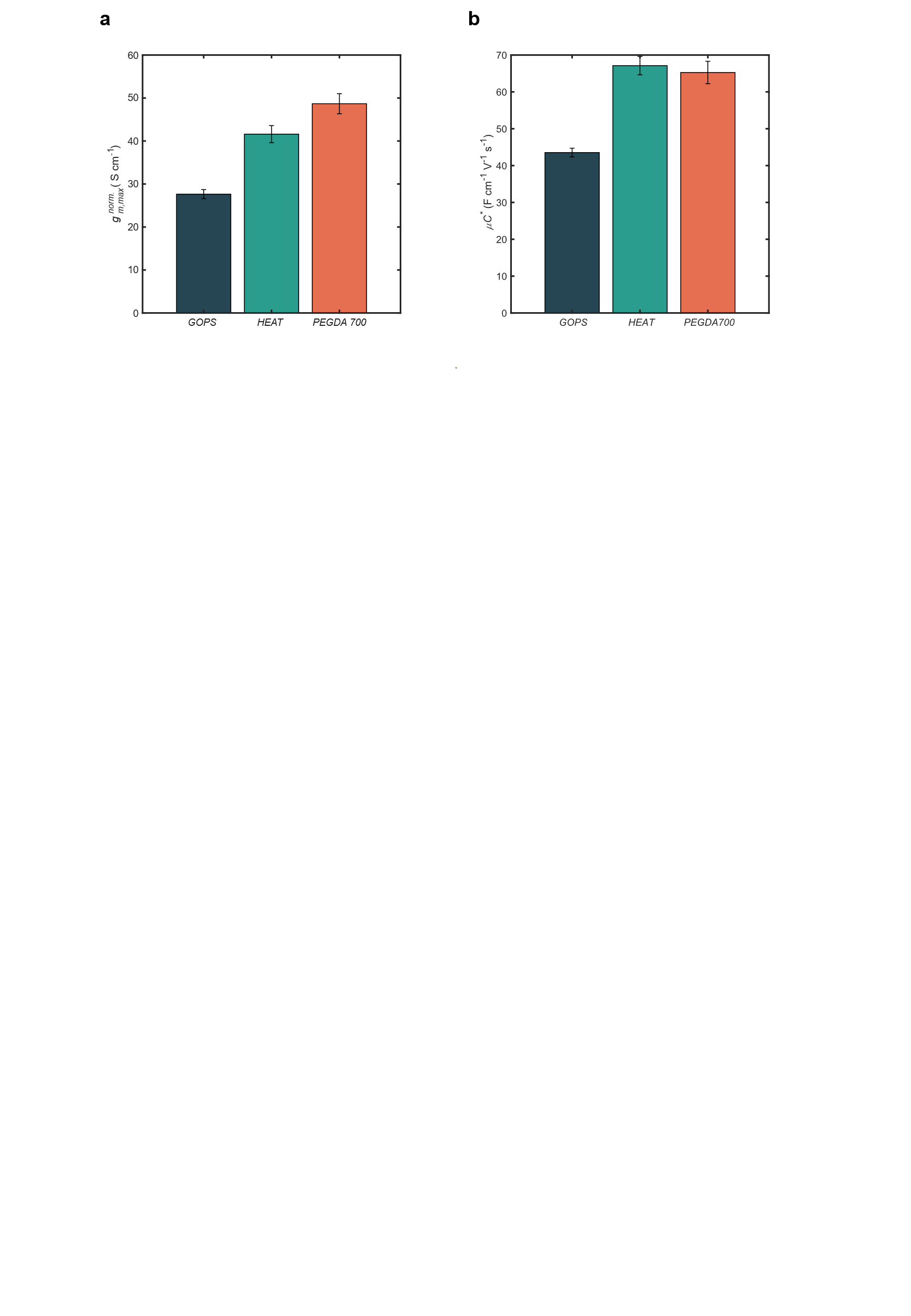


**Figure S9.** Comparison of OECT characteristics for different PEDOT:PSS blends using the swollen thickness. **a)** Maximum transconductance comparison measured from OECT transfer curves in 0.1 M NaCl (*W* = 2000 µm, *L* = 1000 µm). Transconductance is geometry-normalized with a factor of *Ld^-1^W^-1^* and multiplying the thickness (*d*) by the swelling ratio (extracted from AFM measurement, see **Figure S8**). Bar plot shows mean and 95% confidence interval (n = 3). **b)** *µC** comparison measured from the slope of I_DS_^0.5^ in 0.1 M NaCl (*W* = 2000 µm, *L* = 1000 µm) and multiplying the thickness (*d*) by the swelling ratio (extracted from AFM measurement, see **Figure S8**). Bar plot shows mean and 95% confidence interval (n = 3). These metrics follow the same trend compared to the dry state normalization (**Figure 4b** and **4d**).


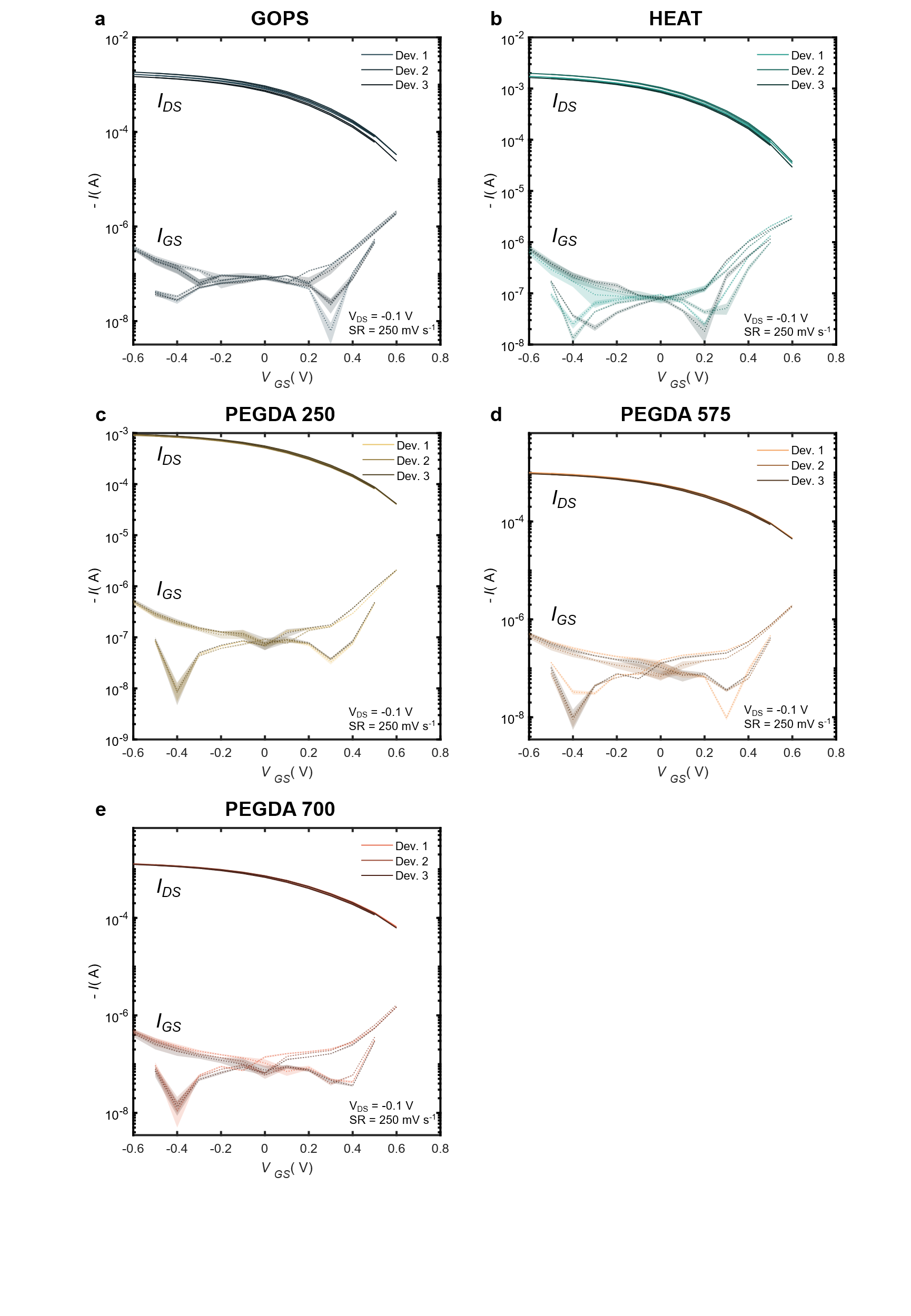


**Figure S10.** Logarithmic transfer curves of 3 different OECTs in 0.1 M NaCl (*W* = 2000 µm, *L* = 1000 µm) with gate current. Mean and standard deviation of 10 cycles are shown for **a)** GOPS blend, **b)** HEAT blend and photo-patterned PEDOT:PSS with **c)** PEGDA 250, **d)** PEGDA 575 and **e)** PEGDA 700
